# Elder Mistreatment and its Subtypes across Different Sociodemographic and Socioeconomic Groups among U.S. Chinese Community-Dwelling Older Adults

**DOI:** 10.23937/2469-5858/1510001

**Published:** 2015-08-26

**Authors:** Ruijia Chen, Xin Qi Dong

**Affiliations:** Rush Institute for Healthy Aging, Rush University Medical Center, USA

**Keywords:** Elder mistreatment subtypes, Chinese older adults, Prevalence, Definition, Culture

## Abstract

**Objectives:**

To examine the risk of overall elder mistreatment (EM) and its subtypes in each sociodemographic and socioeconomic group based on different definitional criteria.

**Methods:**

In person interviews were conducted with 3,159 Chinese older adults in the Greater Chicago Area from 2011 to 2013. Psychological mistreatment, physical mistreatment, sexual abuse, caregiver neglect, and financial exploitation were measured using definitional approaches from the least strict to the strictest criteria.

**Results:**

Physical, psychological mistreatment, and financial exploitation were closely correlated with each other, but caregiver neglect was not correlated with any other types of mistreatment. The risk of EM and its subtypes across sociodemographic groups differed by types and definitions of mistreatment.

**Discussion:**

Future longitudinal studies are needed to quantity the risk and protective factors associated with EM and its subtypes with consideration of definitional issues in Chinese aging populations.

## Introduction

EM was first identified by British gerontologists in 1975 using the term “granny battering”, but it is not until recently that researchers have rigorously examined the issue [1]. Epidemiologic research documents that more than one in ten older adults suffered from some kinds of mistreatment in the U.S [2], making it one of the most significant public health and human rights issues. EM is likely to impose an enormous burden on individual, families, and society [3–7].

Growing interest in EM, though helps elucidate the problem, may at the same time complicate our understanding, given the occurrence of various definitions to assess the issue. Extant research has not yet reached an agreement as to what constitute EM, resulting in various measurements based on different methodological concerns being developed and employed. The divergence in prevalence and characteristics of elder mistreatment has prompted discussion as to whether there is a need for a more unifying measurement. To address this issue, in 2014, Dong used different operational definitions to assess EM and its subtypes and found that the prevalence of EM and its subtypes varied significantly by the strictness of definition used [8]. Yet, no empirical evidence has been presented showing clearly differences in characteristics associated with elder mistreatment by using different operational definitions.

In addition, the majority of existing studies on factors associated with EM tend to regard different EM subtypes as a category and very few studies have analyzed the correlations among different EM subtypes as well as factors associated with each subtype [9,10]. Comijs et al. examined risk factors of verbal aggression, physical aggression, and financial exploitation among 1,797 community-dwelling older adults in the Netherlands and suggested that factors associated with financial exploitation differed from that of verbal and physical aggression [11]. However, the study excluded caregiver neglect, a common type of mistreatment. In a study of 370 elder mistreatment cases, Choi and Mayer found that risk factors of financial exploitation were different from that of physical and psychological mistreatment and/or caregiver neglect. But the study used a case-control design and the number of older adults with physical and mental impairment might be overrepresented [12]. More recently, to compare factors associated with different types of mistreatment, Jackson and Hafemeister analyzed interview and state agency data and found significant differences in risk factors associated with physical abuse, financial exploitation, neglect, and hybrid financial exploitation [13]. However, the study did not include psychological mistreatment and the data mainly came from state agencies. There is a need to more comprehensively understand the correlation among different EM subtypes and to better understand factors associated with each type of mistreatment, through analyzing population-based data.

According to the U.S. Census 2010 estimates, the number of U.S. Chinese older adults aged 60 years and older has been increased to 538,417 [14], most of whom have experienced great cultural and linguistic barriers and disparities in the receipt of health and social services [15]. Moreover, recent research has shown that U.S. Chinese older adults were at high risk for a wide range of psychological issues that may be related to EM and its subtypes [16–18]. There is an urgent need to explore characteristics of EM and its subtypes in this vulnerable population.

This study sought to:

1. Compare the prevalence of EM and its subtypes across different age and gender groups by using different definitions.
2. Explore EM and its subtypes of different definitions across socioeconomic groups.

## Methods

### Population and settings

The Population Study of Chinese Elderly in Chicago (PINE) is a community-engaged, population-based epidemiological study of U.S. Chinese older adults aged 60 and over conducted in Greater Chicago Area. Briefly, the purpose of the PINE study was to collect community-level data of U.S. Chinese older adults to examine the key cultural determinants of health and well-being. The project was initiated by a synergistic community-academic collaboration among the Rush Institute for Healthy Aging, Northwestern University, and many community-based social services agencies and organizations throughout the greater Chicago area.

In order to ensure study relevance to the well-being of the Chinese community and increase community participation, the PINE study implemented extensive culturally and linguistically appropriate community recruitment strategies strictly guided by a community-based participatory research (CBPR) approach [19]. The formation of this community-academic partnership allowed us to develop appropriate research methodology in accordance with the local Chinese cultural context, in which a community advisory board (CAB) plays a pivotal role in providing insights and strategies for conducting research. Board members were community stakeholders and residents enlisted through over twenty civic, health, social and advocacy groups, community centers and clinics in the city and suburbs of Chicago. The board works extensively with investigative team to develop and examine study instrument to ensure cultural sensitivity and appropriateness.

### Study design and procedure

The research team implemented a targeted community-based recruitment strategy by first engaging community centers as our main recruitment sites throughout the greater Chicago area. Over twenty social services agencies, community centers, health advocacy agencies, faith-based organizations, senior apartments and social clubs served as the basis of study recruitment sites. Community-dwelling older adults who aged 60 years and over and self-identified as Chinese were eligible to participant in the study. Out of 3,439 eligible older adults approached, 3,159 agreed to participate in the study, yielding a response rate of 91.9 %. More in-depth details of the PINE study design have been published elsewhere [20].

In order to ensure cultural and linguistic sensitivity, trained multicultural and multi-lingual interviewers conducted face-to-face home interviews with participants in their preferred language and dialects, such as English, Cantonese, Taishanese, Mandarin or Teochew dialect. To protect participant confidentiality, the interviews were conducted in a private area of the participant’s house. Data were collected using state-of-science innovative web-based software which recorded simultaneously in English, Chinese traditional and simplified characters. Based on the available census data drawn from U.S. Census 2010 and a random block census project conducted in the Chinese community in Chicago, the PINE study is representative of the Chinese aging population in the greater Chicago area [21]. The study was approved by the Institutional Review Boards of the Rush University Medical Center.

### Measurements

#### Sociodemographic and socioeconomic characteristics

Basic sociodemographic and socioeconomic information collected included age (years), gender, education (years), and annual personal income.

#### EM subtypes

EM subtypes were assessed by a 56-item self-report measure that captured the following subtypes: psychological mistreatment, physical mistreatment, sexual abuse, caregiver neglect, and financial exploitation. The measurement has shown great validity in prior research among Chinese populations [22]. Content validity was assessed by a group of bilingual and bicultural study researchers and prominent members from the Chinese community with expertise in Chinese cultural health and aging issues. Questions of each EM subtype have been published elsewhere [8].

#### Psychological mistreatment

Psychological mistreatment is the infliction of anguish, pain, or distress through verbal or nonverbal acts. It includes but is not limited to teasing, insulting, and threatening [23]. Five definitions, ranging from less strict to more strict, were constructed for psychological mistreatment: 1) an affirmative “yes” response to having experienced any of the eight CTS psychological mistreatment items (Psych-1); 2) affirmative responses in two or more items (Psych -2); 3) affirmative responses in three or more items (Psych-3); 4) affirmative responses in three or more items *or* threats to send to nursing home or abandonment (Psych_Beach) [24]; and 5) affirmative responses in 10+ times for CTS items (Psych_Pillemer) [25].

#### Physical mistreatment

Physical mistreatment is the non-accidental infliction of physical force that causes a bodily injury, pain or impairment, which may include hitting, shocking, pushing, and kicking [23]. Inconsistencies in operational definitions are more often observed when it comes to defining psychological mistreatment, caregiver neglect and financial exploitation, but are less likely to occur when defining sexual abuse and physical mistreatment, which are more straightforward; therefore, we only used one definition to assess physical mistreatment and sexual abuse. We used 10 items in the CTS to assess physical mistreatment. Any positive answer to the 10 items was treated as having physical mistreatment.

#### Sexual abuse

Sexual abuse refers to non-consensual sexual contact of any kind with an elderly person [23]. Sexual abuse in this study was measured by a single criterion that was derived from the CTS. We asked participants if they had been touched in private areas when they did not want to be. Any positive answer to the item was treated as having experienced sexual abuse.

#### Financial exploitation

Financial exploitation is the illegal or improper use of an elder’s funds, property, or assets [23]. For financial exploitation, we used two different definitions: 1) any positive answer on the 17-item measure (financial-1), and 2) any positive answer on the 14-item measure, excluding three items (felt entitled to use your money, prevented you from spending your money, and tricked or pressured you into buying something) that may be less likely to be considered exploitative (financial-2).

#### Caregiver neglect

Caregiver neglect is defined as the refusal or failure to fulfill any part of a person’s obligations or duties to an elder [23]. In this study, we used a 20-item unmet needs assessment to measure caregiver neglect [8]. Participants were also asked to evaluate the severity of their unmet needs (no/mild/moderate/severe). We used two different operational definitions: 1) any unmet needs + living with a family member (neglect-1), and 2) moderate/severe unmet needs + living with a family member (neglect-2).

#### Overall EM

According to the above definitions of EM subtypes, three definitions based on different levels of strictness were used for defining overall EM: 1)broadly-defined overall EM: psych-1, physical, sexual, neglect-1, and financial-1; 2)moderately-defined overall EM: physical, sexual, and varying levels of neglect, psychological mistreatment and financial exploitation; and 3) strictly-defined overall EM: psych_Pillemer, physical, sexual, neglect-2, and financial-2.

### Data analysis

Pearson correlation coefficients were calculated to examine the correlation among subtypes of mistreatment. Logistic regression analyses were then conducted to compare the prevalence of EM and its subtypes among different groups of age (group 1: 60–70 years old; group 2: 71–80 years old; group 3: >80 years old), gender (women vs. men), education (group 1: 0–8 years; group 2: 9–12 years; group 3: >12 years), and annual income (group 1: <5K; group 2: 5–10 K; group 3: >10 K). All statistical analyses were undertaken using SAS, Version 9.2 (SAS Institute Inc., Cary, NC).

## Results

### Correlations among different subtypes and definitions of EM

In total, 3,159 Chinese older adults participated in the study, of which 58.9% were women and the average age was 72.8 years (*SD*=8.3, range=60–105). The correlations among different types of mistreatment are presented in

Table 1 Physical mistreatment was correlated with all definitions of psychological mistreatment (r ranged from 023–0.38, p<0.001) and financial exploitation (r financial_1=0.05, r financial_2=0.06, p<0.01), but not with sexual abuse and caregiver neglect. Likewise, psychological mistreatment was correlated with physical and financial exploitation (r ranged from 0.07–0.14, p<0.001), but not with sexual abuse and caregiver neglect. Financial exploitation was correlated with physical, sexual abuse (r financial_1=0.04, r financial_2=0.04, p<0.05) and psychological mistreatment, but not with caregiver neglect.

### EM and its subtypes across different age groups

Table 2 shows the prevalence of EM and its subtypes in each age group. Older adults aged 60 to 70 years old had a higher prevalence of all definitions of overall EM than other age groups. Regarding psychological mistreatment, the oldest age group had the highest prevalence of the least strict psychological mistreatment. In terms of caregiver neglect, the oldest age group also reported the highest prevalence of caregiver neglect.

### EM and its subtypes across different gender groups

Table 3 presents the prevalence of EM and its subtypes among men and women. Men reported a higher prevalence of the strictly-defined EM and all definitions of financial exploitation compared to women. Women were 1.42 times more likely to experience the least strict psychological mistreatment than men. No significant gender differences were found in physical mistreatment and caregiver neglect.

### EM and its subtypes across different socioeconomic groups

Table 4 presents the prevalence of EM by education and income levels. Older adults in the lowest education group reported lower prevalence of overall EM than other education groups. As for psychological mistreatment, the “>12 years” group had a higher prevalence than the “0–8 years” group. Older adults in the lowest education group were at the lowest risk of financial exploitation. No significant differences in prevalence of physical mistreatment were found among different educational groups.

Regarding income, older adults with an annual income of 5–10K and less than 5K were less likely to experience the strictly-defined overall EM than those earning 10K and more. Similarly, for financial exploitation, older adults with an annual income of 5–10K and 5K and less were at lower risk than those who earned 10K and more. No significant differences in psychological mistreatment were found among the income groups.

## Discussion

This study explored EM and its subtypes of different operational definitions among 3,159 community-dwelling U.S. Chinese older adults. The findings demonstrate that physical mistreatment, psychological mistreatment, and financial exploitation were correlated with each other, but caregiver neglect was not correlated with other EM subtypes. Sociodemographic and socioeconomic characteristics of EM differed by subtypes and definitions.

In this study, physical mistreatment, psychological mistreatment, and financial exploitation were significantly correlated with each other, suggesting that older adults may be victimized by multiple types of mistreatment at the same time. However, caregiver neglect was not correlated with other EM subtypes. This may be explained by differences in the nature of EM subtypes. Caregiver neglect can be either intentional or unintentional. Strasser and Fulmer described unintentional caregiver neglect as “the inadvertent action resulting in harm to an elderly person usually due to ignorance, inexperience, or lack of caregiver ability/desire to provide proper care” [26]. Unintentional neglect may occur when the caregiver lacks of resources and knowledge or is overburdened. This is often true in immigrant families where adult children may lack appropriate care giving knowledge and skills, lack awareness regarding available resources and support due to language or cultural barriers, or be unable to provide due to excessive time commitments and strenuous physical labor. Unlike caregiver neglect, other types of mistreatment such as physical mistreatment, psychological mistreatment, and financial exploitation tend to be caused by intentional acts. Thus, caregiver neglect, especially unintentional neglect, is more likely to occur as a single form of mistreatment as compared to other mistreatment types.

Socio-demographic characteristics associated with EM varied by definitions and types of EM. Regarding age, older adults in the youngest age group faced the lowest risk for broadly-defined and strictly-defined overall EM, but this trend was not observed when using the moderate definition. Older adults at the oldest age group had the lowest risk of the least strict psychological mistreatment only, while no age differences were found in other psychological mistreatment definitions. With respect to gender, although many prior studies suggested a higher prevalence of EM among women than men [27,22], this study showed that men were at greater risk for strictly-defined overall EM and for both definitions of financial exploitation. It may be that Chinese men tend to be the family breadwinner and household head and are more likely to manage household finances, which in turn, may predispose them to financial exploitation. For psychological mistreatment, women were only at greater risk using the least strict psychological mistreatment. In addition, no differences in prevalence of caregiver neglect were found in men and women, contrasting the finding of a study in Chinese older adults in mainland China that men were more likely to be neglected [28]. These findings altogether challenge the traditional notion that women are at higher risk for EM and show that operational definitions play important roles of determining socio-demographics characteristics of EM.

With respect to socioeconomic characteristics, this study suggests that lowest educated older adults had the lowest prevalence of moderately-defined EM and all definitions of financial exploitation, which is consistent with a study, found the prevalence of fraud was more commonly reported among higher educated older adults [29]. As for income, older adults with the highest income levels had the highest prevalence of strictly-defined overall EM and all definitions of financial exploitation. We postulate that older adults with higher income levels may be more involved in managing finances, which may increase their risk for exploitation. No differences in prevalence of caregiver neglect were found among different income groups, which contradict prior studies suggesting that low income contributed to elder neglect [2,12].

The interpretation of the study findings should consider various limitations. First, this study only examined Chinese populations in the greater Chicago area, and therefore the findings may not be generalizable to Chinese aging populations in other areas. Second, this study only included a select set of definitions and we may have excluded other representative definitions. Third, this study did not distinguish between older adults with and without intact cognitive function. We suspect that physical mistreatment, psychological mistreatment, financial exploitation, and neglect may be more likely to coexist with among older adults with cognitive impairment. Furthermore, after thoughtful consideration, this study did not examine the prevalence of sexual abuse among different sociodemographic groups because of the small number of people reporting having been sexually mistreated. Finally, this study was designed as a cross-sectional study. Future longitudinal studies should be conducted to better examine the findings.

When we take these limitations into account, this study has important research and policy implications. This study emphasizes that different definitions may lead to different characteristics associated with overall mistreatment and its subtypes. Research in EM should expand effort to explore other risk factors associated with EM using different definitions and to develop potential approaches to address the issues of inconsistencies in definitions. The variation of characteristics associated with EM subtypes also implies that community organizations should tailor intervention programs and services to the types of the mistreatment under the specific cultural context. In particular, caregiver and self-neglect comprise the largest category of cases reported to Adult Protective Services; because the present study shows that caregiver neglect was not correlated with other types of mistreatment, special intervention and prevention efforts such as reducing caregiver burden and promoting home care services should be geared toward caregiver neglect.

## Conclusion

In sum, our study shows that EM and its subtypes across different socio-demographic groups differed by types and definitions of mistreatment. Further Studies should continue to explore risk factors of EM and its subtypes using different definitions and to develop a sound and consistent definition. This study also underscores the need to tailor intervention and prevention to address specific subtypes of EM.

## Figures and Tables

**Table 1:** Correlations among different subtypes and definitions of elder mistreatment in a Chinese population

	EM Broad	EM Mod	EM Strict	Physical	Sexual	Psych 1	Psych 2	Psych 3	Psych Beach	Psych Pillemer	Financial_1	Financial_2	Neglect 1	Neglect 2
**EM Broad**	1.0													
**EM Moderate**	0.78+	1.0												
**EM Strict**	0.68+	0.88+	1.0											
**Physical**	0.17+	0.23+	0.26+	1.0										
**Sexual**	0.07+	0.10+	0.11+	−0.01	1.0									
**Psych_1**	0.56+	0.42+	0.21+	0.23+	0.03	1.0								
**Psych_2**	0.40+	0.52+	0.23+	0.29+	−0.01	0.72+	1.0							
**Psych_3**	0.28+	0.36+	0.24+	0.38+	−0.01	0.49+	0.69+	1.0						
**Psych_Beach**	0.29+	0.37+	0.23+	0.36+	−0.01	0.52+	0.70+	0.94+	1.0					
**Psych_Pillemer**	0.18+	0.22+	0.26+	0.32+	−0.01	0.32+	0.41+	0.39+	0.37+	1.0				
**Financial_1**	0.54+	0.70+	0.75+	0.05#	0.04*	0.14+	0.13+	0.13+	0.13+	0.07+	1.0			
**Financial_2**	0.53+	0.68+	0.77+	0.06#	0.04*	0.14+	0.13+	0.13+	0.14+	0.08+	0.97+	1.0		
**Neglect_1**	0.59+	0.28+	0.32+	−0.01	−0.02	−0.01	−0.01	0.01	0.01	−0.01	0.01	0.01	1.0	
**Neglect_2**	0.37+	0.48+	0.54+	0.01	−0.01	0.03	0.01	0.04	0.03	0.02	0.03	0.03	0.62+	1.0

* p <0.05,

# p< 0.01,

+ p<0.001

**Notes:** EM_Broad: broadly-defined EM; EM_Moderate: moderately-defined EM; EM_strict: strictly-defined EM; EM. Psych_1: an affirmative “yes” response to having experienced any of the eight CTS psychological mistreatment items; Psych_2: affirmative responses in two or more items (Psych -2); Psych_3: affirmative responses in three or more items (Psych-3); Psych_Beach: affirmative responses in three or more items or threats to send to nursing home or abandonment; Psych_Pillemer: affirmative responses in 10+ times for CTS items; Financial _1: any positive answer on the 17-item measure (financial-1); Financial _2: any positive answer on the 14-item measure, but excluding three items that may be less likely to be considered as financial exploitation: felt entitled to use your money, prevented you from spending your money, and tricked or pressured you into buying something (financial-2); neglect: 1) any unmet needs + living with a family member (neglect-1), and 2) moderate/severe unmet needs + living with a family member (neglect-2).

**Table 2:** Elder Mistreatment and its subtypes across different age groups

	Group1: 60–70 (yrs) % (95% CI)	Group 2: 71–80 (yrs) % (95% CI)	Group 3: > 80 (yrs) % (95% CI)	Group 2 vs. Group 1 OR (95% CI)	Group 3 vs. Group 1 OR (95% CI)
**EM _ Broad**	22.15 (19.91–24.38)	29.86 (27.24–32.49)	26.12 (22.79–29.45)	1.62 (1.35–1.94)*	1.26 (1.02–1.56)+
**EM _ Strict**	11.41 (9.70–13.13)	16.09 (13.98–18.21)	15.07 (12.37–17.78)	1.53 (1.22–1.93)*	1.37 (1.05–1.78)+
**EM _ Moderate**	14.81 (12.90–16.73)	20.05 (17.75–22.35)	16.57 (13.75–19.38)	1.52 (1.24–1.88)*	1.15 (0.89–1.48)
**Physical**	0.91 (0.40–1.42)	1.12 (0.51–1.73)	1.20 (0.37–2.02)	1.30 (0.59–2.87)	1.34 (0.55–3.25)
**Psychological_1**	9.77 (8.17–11.37)	11.65 (9.80–13.49)	6.95 (4.71–8.47)	1.33 (1.03–1.71)+	0.67 (0.47–0.95)+
**Psychological_2**	5.23 (4.03–6.43)	6.47 (5.05–7.89)	3.44 (2.06–4.83)	1.36 (0.97–1.90)	0.66 (0.41–1.07)
**Psychological_3**	2.42 (1.59–3.25)	2.76 (1.82–3.70)	2.54 (1.35–3.74)	1.03 (0.62–1.70)	0.99 (0.56–1.78)
**Psychological_ Beach**	2.58 (1.72–3.43)	3.28 (2.25–4.30)	2.84 (1.58–4.10)	1.19 (0.75–1.92)	1.06 (0.61–1.84)
**Psychological_Pillemer**	0.83 (0.34–1.32)	1.81 (1.04–2.58)	0.30 (0.00–0.71)	1.83 (0.90–3.69)	0.31 (0.07–1.36)
**Financial _ 1**	8.88 (7.34–10.41)	10.26 (8.51–12.00)	8.23 (6.15–10.32)	1.25 (0.96–1.64)	0.94 (0.68–1.31)
**Financial _ 2**	8.35 (6.85–9.84)	9.66 (7.96–11.35)	8.23 (6.15–10.32)	1.24 (0.94–1.63)	1.00 (0.72–1.39)
**Neglect_ 1**	7.02 (5.63–8.41)	12.39 (10.44–14.33)	18.06 (14.91–21.20)	2.02 (1.54–2.67)*	2.94 (2.19–3.95)*
**Neglect _ 2**	2.39 (1.56–3.22)	5.37 (4.04–6.71)	7.99 (5.77–10.20)	2.26 (1.47–3.48)*	3.34 (2.14–5.22)*

CI: 95% Confidence interval is based on the inversion of Wald test constructed with the use of standard errors.

+ p<0.05,

* p<0.005

EM_Broad: broadly-defined EM; EM_Moderate: moderately-defined EM; EM_strict: strictly-defined EM; Psych_1: an affirmative “yes” response to having experienced any of the eight CTS psychological mistreatment items; Psych_2: affirmative responses in two or more items (Psych -2); Psych_3: affirmative responses in three or more items (Psych-3); Psych_Beach: affirmative responses in three or more items or threats to send to nursing home or abandonment; Psych_Pillemer: affirmative responses in 10+ times for CTS items; Financial _1: any positive answer on the 17-item measure (financial-1); Financial _2: any positive answer on the 14-item measure, but excluding three items that may be less likely to be considered as financial exploitation: felt entitled to use your money, prevented you from spending your money, and tricked or pressured you into buying something (financial-2); neglect: 1) any unmet needs + living with a family member (neglect-1), and 2) moderate/severe unmet needs + living with a family member (neglect-2).

**Table 3:** Gender and elder mistreatment subtypes across different definitions

	Group1: Men % (95% CI)	Group 2: Women % (95% CI)	Women vs. Men OR (95% CI)
**EM _ Broad**	26.57 (24.19–28.94)	25.30 (23.31–27.29)	0.94 (0.79–1.10)
**EM _ Strict**	15.77 (13.81–17.74)	12.57 (11.05–14.09)	0.77 (0.63–0.94)+
**EM _ Moderate**	18.64 (16.54–20.74)	16.01 (14.33–17.69)	0.83 (0.69–1.00)
**Physical**	0.98 (0.45–1.52)	1.09 (0.62–1.57)	1.11 (0.55–2.25)
**Psychological_1**	8.02 (6.55–9.48)	11.07 (9.63–12.51)	1.42 (1.12–1.83)+
**Psychological_2**	4.39 (3.28–5.49)	5.97 (4.89–7.06)	1.38 (0.99–1.92)
**Psychological_3**	2.27 (1.47–3.07)	2.79 (2.04–3.55)	1.24 (0.78–1.96)
**Psychological_ Beach**	2.50 (1.66–3.34)	3.18 (2.37–3.98)	1.28 (0.83–1.98)
**Psychological_Pillemer**	0.76 (0.29–1.22)	1.31 (0.79–1.83)	1.75 (0.83–3.66)
**Financial _ 1**	11.66 (9.93–13.39)	7.51 (6.30–8.72)	0.62 (0.48–0.78)*
**Financial _ 2**	11.13 (9.43–12.82)	7.12 (5.94–8.30)	0.61 (0.48–0.78)*
**Neglect_ 1**	11.45 (9.70–13.21)	10.92 (9.43–12.40)	0.95 (0.75–1.19)
**Neglect _ 2**	4.98 (3.78–6.17)	4.28 (3.32–5.25)	0.86 (0.61–1.21)

CI: 95% Confidence interval is based on the inversion of Wald test constructed with the use of standard errors.

+ p<0.05,

* p<0.001

Notes: EM_Broad: broadly-defined EM; EM_Moderate: moderately-defined EM; EM_strict: strictly-defined EM; Psych_1: an affirmative “yes” response to having experienced any of the eight CTS psychological mistreatment items; Psych_2: affirmative responses in two or more items (Psych -2); Psych_3: affirmative responses in three or more items (Psych-3); Psych_Beach: affirmative responses in three or more items *or* threats to send to nursing home or abandonment; Psych_Pillemer: affirmative responses in 10+ times for CTS items; Financial _1: any positive answer on the 17-item measure (financial-1); Financial _2: any positive answer on the 14-item measure, but excluding three items that may be less likely to be considered as financial exploitation: felt entitled to use your money, prevented you from spending your money, and tricked or pressured you into buying something (financial-2); neglect: 1) any unmet needs + living with a family member (neglect-1), and 2) moderate/severe unmet needs + living with a family member (neglect-2).

**Table 4:** Socioeconomic status and elder mistreatment subtypes across different definitions

	Education 1: 0–8 (yrs) % (95% CI)	Education 2: 9–12 (yrs) % (95% CI)	Education 3: > 12 (yrs) % (95% CI)	Group 1. Vs. Group 2 OR (95% CI)	Group 1 vs. Group 3 OR (95% CI)
**EM _ Broad**	21.81 (19.74–23.88)	27.34 (24.49–30.19)	32.88 (29.30–36.46)	0.77 (0.62–0.95)+	0.57 (0.47–0.69)*
**EM _ Strict**	10.22 (8.71–11.74)	15.85 (13.52–18.19)	19.61 (16.59–22.63)	0.77 (0.59–1.00)	0.47 (0.36–0.60)*
**EM _ Moderate**	12.50 (10.85–14.15)	19.04 (16.53–21.55)	24.89 (21.60–28.18)	0.71 (0.56–0.90)+	0.43 (0.34–0.54)*
**Physical**	0.65 (0.25–1.05)	1.60 (0.80–2.40)	1.21 (0.38–2.04)	1.33 (0.56–3.15)	0.54 (0.21–1.37)
**Psychological_1**	6.85 (5.58–8.11)	10.55 (8.59–12.52)	15.43 (12.68–18.19)	0.65 (0.48–0.87)*	0.40 (0.30–0.54)*
**Psychological_2**	3.33 (2.43–4.22)	5.97 (4.45–7.49)	8.93 (6.75–11.10)	0.65 (0.44–0.95)+	0.35 (0.24–0.52)*
**Psychological_3**	1.89 (1.21–2.57)	2.56 (1.55–3.57)	4.24 (2.70–5.77)	0.59 (0.34–1.03)	0.44 (0.26–0.74)*
**Psychological_ Beach**	2.15 (1.43–2.88)	2.77 (1.72–3.82)	4.84 (3.20–6.48)	0.56 (0.33–0.95)+	0.43 (0.26–0.71)*
**Psychological_Pillemer**	0.52 (0.16–0.88)	1.38 (0.64–2.13)	1.96 (0.91–3.02)	0.70 (0.32–1.52)	0.26 (0.11–0.63)*
**Financial _ 1**	5.68 (4.57–6.84)	10.76 (8.77–12.74)	15.58 (12.82–18.35)	0.65 (0.49–0.88)*	0.33 (0.24–0.44)*
**Financial _ 2**	5.16 (4.05–6.26)	10.44 (8.48–12.39)	15.13 (12.40–17.86)	0.65 (0.49–0.88)+	0.31 (0.22–0.42)*
**Neglect_ 1**	13.08 (11.33–14.84)	10.17 (8.28–12.13)	8.01 (5.90–10.11)	1.30 (0.91–1.86)	1.73 (1.25–2.39)*
**Neglect _ 2**	4.95 (3.82–6.08)	4.09 (2.80–5.38)	4.08 (2.55–5.62)	1.00 (0.60–1.67)	1.22 (0.77–1.94)
	**Income 1: < 5K** **% (95% CI)**	**Income 2: 5–10K** **% (95% CI)**	**Income 3: >10K** **% (95% CI)**	**Group 2 vs Group 3** **OR (95% CI)**	**Group 1 vs Group 3** **OR (95% CI)**
**EM _ Broad**	26.06 (23.39–28.73)	25.54 (23.42–27.67)	26.45 (22.44–30.46)	0.95 (0.75–1.21)	0.98 (0.76–1.26)
**EM _ Strict**	13.17 (11.12–15.23)	13.11 (11.47–14.76)	17.85 (14.37–21.33)	0.69 (0.53–0.92)+	0.69 (0.52–0.94)+
**EM _ Moderate**	16.83 (14.55–19.10)	16.33 (14.53–18.13)	20.22 (16.56–23.87)	0.77 (0.59–1.00)	0.79 (0.60–1.06)
**Physical**	1.73 (0.94–2.52)	0.43 (0.11–0.75)	1.51 (0.40–2.61)	0.29 (0.10–0.82)+	1.15 (0.48–2.78)
**Psychological_1**	11.26 (9.34–13.18)	8.87 (7.48–10.26)	9.46 (6.80–12.12)	0.93 (0.65–1.33)	1.21 (0.84–1.75)
**Psychological_2**	6.54 (5.04–8.05)	4.78 (3.74–5.82)	4.52 (2.63–6.40)	1.06 (0.65–1.74)	1.48 (0.89–2.45)
**Psychological_3**	3.46 (2.35–4.58)	2.11 (1.41–2.81)	2.15 (0.83–3.47)	0.98 (0.48–2.00)	1.63 (0.80–3.32)
**Psychological_ Beach**	3.85 (2.68–5.02)	2.42 (1.67–3.17)	2.37 (0.98–3.75)	1.02 (0.52–2.01)	1.65 (0.84–3.25)
**Psychological_Pillemer**	1.73 (0.90–2.53)	0.74 (0.32–1.16)	0.86 (0.02–1.70)	0.86 (0.28–2.69)	2.03 (0.68–6.04)
**Financial _ 1**	9.15 (7.40–10.91)	8.12 (6.78–9.45)	13.55 (10.44–16.66)	0.56 (0.41–0.78)*	0.64 (0.46–0.90)+
**Financial _ 2**	8.38 (6.70–10.07)	7.87 (6.56–9.18)	13.12 (10.05–16.19)	0.57 (0.41–0.78)*	0.61 (0.43–0.86)*
**Neglect_ 1**	9.82 (8.04–11.84)	13.03 (11.32–14.74)	7.89 (5.42–10.37)	1.74 (1.20–2.54)*	1.27 (0.85–1.89)
**Neglect _ 2**	3.51 (2.37–4.65)	5.31 (4.17–6.44)	4.17 (2.33–6.00)	1.29 (0.77–2.15)	0.84 (0.47–1.48)

CI: 95% Confidence interval is based on the inversion of Wald test constructed with the use of standard errors.

+ p<0.05,

* p<0.005.

**Notes:** EM_Broad: broadly-defined EM; EM_Moderate: moderately-defined EM; EM_strict: strictly-defined EM; Psych_1: an affirmative “yes” response to having experienced any of the eight CTS psychological mistreatment items; Psych_2: affirmative responses in two or more items (Psych -2); Psych_3: affirmative responses in three or more items (Psych-3); Psych_Beach: affirmative responses in three or more items *or* threats to send to nursing home or abandonment; Psych_Pillemer: affirmative responses in 10+ times for CTS items; Financial _1: any positive answer on the 17-item measure (financial-1); Financial _2: any positive answer on the 14-item measure, but excluding three items that may be less likely to be considered as financial exploitation: felt entitled to use your money, prevented you from spending your money, and tricked or pressured you into buying something (financial-2); neglect: 1) any unmet needs + living with a family member (neglect-1), and 2) moderate/severe unmet needs + living with a family member (neglect-2).

## References

[1] Burston GR (1975). Granny battering. Br Med J.

[2] Acierno R, Hernandez MA, Amstadter A, Resnick HS, Steve K (2010). Prevalence and correlates of emotional, physical, sexual, and financial abuse and potential neglect in the United States: The National Elder Mistreatment Study. Am J Public Health.

[3] Begle AM, Strachan M, Cisler JM, Amstadter AB, Hernandez M (2011). Elder Mistreatment and Emotional Symptoms Among Older Adults in a Largely Rural Population: The South Carolina Elder Mistreatment Study. J Interpers Violence.

[4] Dong X, Simon M, Mendes de Leon C, Fulmer T, Beck T (2009). Elder Self-neglect and Abuse and Mortality Risk in a Community-Dwelling Population. JAMA.

[5] Dong X, Simon MA, Evans D (2012). Prospective study of the elder self-neglect and ED use in a community population. Am J Emerg Med.

[6] Dong X, Simon MA (2013). Elder abuse as a risk factor for hospitalization in older persons. JAMA Intern Med.

[7] Dong X, Simon MA (2013). Association between reported elder abuse and rates of admission to skilled nursing facilities: findings from a longitudinal population-based cohort study. Gerontology.

[8] Dong X (2014). Do the Definitions of Elder Mistreatment Subtypes Matter? Findings From the PINE Study. The Journals of Gerontology Series A Biological Sciences and Medical Sciences.

[9] Chokkanathan S (2014). Factors associated with elder mistreatment in rural Tamil Nadu, India: a cross-sectional survey. International Journal of Geriatric Psychiatry.

[10] Perez-Rojo G, Izal M, Montorio I, Penhale B (2009). Risk factors of elder abuse in a community dwelling Spanish sample. Archives of gerontology and geriatrics.

[11] Comijs HC, Smit JH, Pot AM, Bouter LM, Jonker C (1999). Risk indicators of elder mistreatment in the community. Journal of Elder Abuse & Neglect.

[12] Choi NG, Kim J, Asseff J (2009). Self-neglect and neglect of vulnerable older adults: reexamination of etiology. Journal of Gerontological Social Work.

[13] Jackson SL, Hafemeister TL (2011). Risk factors associated with elder abuse: The importance of differentiating by type of elder maltreatment. Violence Vict.

[14] United States Census Bureau (2010). American Fact Finder.

[15] Mui AC, Kang SY, Kang D, Domanski MD (2007). English language proficiency and health-related quality of life among Chinese and Korean immigrant elders. Health social work.

[16] Dong X, Chen R, Chang E-S, Simon MA (2014). The prevalence of suicide attempts among community-dwelling US Chinese older adults: findings from the PINE study. Ethnicity and Inequalities in Health and Social Care.

[17] Dong X, Chen R, Li C, Simon MA (2014). Understanding Depressive Symptoms Among Community-Dwelling Chinese Older Adults in the Greater Chicago Area. J Aging Health.

[18] Dong X, Chen R, Wong E, Simon MA (2014). Suicidal ideation in an older US Chinese population. J Aging Health.

[19] Dong X, Chang ES, Simon M, Wong E (2011). Sustaining Community-University Partnerships: Lessons learned from a participatory research project with elderly Chinese. Gateways: International Journal of Community Research and Engagement.

[20] Dong X, Wong E, Simon MA (2014). Study design and implementation of the PINE study. J Aging Health.

[21] Simon M, Chang ES, Rajan KB, Welch M, Dong X (2014). Demographic characteris of U.S. Chinese Older Adults in the Greater Chicago Area: Assessing the representativeness of the PINE study. J Aging Health.

[22] Dong X, Simon MA, Gorbien M (2007). Elder abuse and neglect in an urban Chinese population. Journal of Elder Abuse & Neglect.

[23] National Center on Elder Abuse (2014). Types of Abuse.

[24] Beach SR, Schulz R, Castle NG, Rosen J (2010). Financial exploitation and psychological mistreatment among older adults: Differences between African Americans and Non-African Americans in a population-based survey. Gerontologist.

[25] Pillemer K, Finkelhor D (1988). The prevalence of elder abuse: A random sample survey. The Gerontologist.

[26] Strasser SM, Fulmer T (2007). The clinical presentation of elder neglect: what we know and what we can do. Journal of the American Psychiatric Nurse Association.

[27] Chokkanathan S, Lee AE (2006). Elder mistreatment in urban India: A community based study. J Elder Abuse Negl.

[28] Wu L, Chen H, Hu Y, Xiang H, Yu X (2012). Prevalence and associated factors of elder mistreatment in a rural community in People’s Republic of China: a cross-sectional study. PloS one.

[29] Lichtenberg PA, Stickney L, Paulson D (2013). Is Psychological Vulnerability Related to the Experience of Fraud in Older Adults?. Clin Gerontol.

